# Evaluating urban community knowledge, attitudes, and preventive actions (KAP) using the health belief model: Insights from an ideation-informed interpretation of recurrent dengue outbreaks in Selangor, Malaysia

**DOI:** 10.1371/journal.pntd.0013545

**Published:** 2025-10-10

**Authors:** Nurul Adilah Samsudin, Zul-‘Izzat Ikhwan Zaini, Ching Sin Siau, Hidayatulfathi Othman

**Affiliations:** 1 Centre for Toxicology and Health Risk Studies (CORE), Faculty of Health Sciences, Universiti Kebangsaan Malaysia, Jalan Raja Muda A. Aziz, Kuala Lumpur, Malaysia; 2 Department of Basic Science, Faculty of Health Sciences, Universiti Teknologi Mara (UiTM), Cawangan Pulau Pinang, Kampus Bertam, Kepala Batas, Penang, Malaysia; 3 Centre for Community Health Studies (ReaCH), Faculty of Health Sciences, Universiti Kebangsaan Malaysia, Jalan Raja Muda A. Aziz, Kuala Lumpur, Malaysia; Kenya Agricultural and Livestock Research Organization, KENYA

## Abstract

**Background:**

Dengue fever remains endemic in Malaysia and presents a persistent public health concern, affecting Selangor in particular. There is an information gap regarding the knowledge, attitudes, and practices (KAP) of residents in Hulu Langat, Selangor, regarding dengue infection. This study aimed to clarify the dengue-related KAP of Hulu Langat residents using the health belief model (HBM) and discuss the potential integration of ideation concepts to provide a more comprehensive understanding.

**Materials and methods:**

A community‑based cross‑sectional survey was conducted among 146 individuals in Hulu Langat during March 2022. Data on the respondents’ sociodemographic details and dengue KAP were obtained using an HBM-based semi-structured questionnaire. The data were analyzed using descriptive statistics, the Kaiser-Meyer-Olkin (KMO) measure, factor analysis, Student’s *t*-test, and Spearman’s rank correlation coefficient.

**Results:**

Among the 146 participants, only 38.7% demonstrated sufficient overall knowledge regarding dengue, which included knowledge about Aedes mosquitoes and dengue disease, as well as knowledge of mosquito breeding sites and prevention measures. Notably, the proportion of participants with a positive attitude was relatively low at 27.3%, and only 42.5% demonstrated good preventive practices against Aedes mosquito breeding and dengue fever. Correlation analysis showed a negligible relationship between knowledge and attitude, a significant negative correlation between knowledge and practice, and a moderate negative correlation between attitude and practice. These results highlight the complex dynamics influencing dengue prevention behaviors in the studied population.

**Conclusion:**

The results revealed that the respondents had notable KAP gaps related to preventing *Aedes* mosquito breeding and dengue fever. The results demonstrated that raising awareness, shaping a positive attitude, and promoting effective prevention behavior in the studied population require improvement. These results underscore the importance of targeted interventions and comprehensive public health strategies to enhance understanding, attitudes, and practices regarding dengue prevention among the community, especially in recurrent hotspot areas.

## Author summary

Dengue fever remains a major public health concern in Malaysia, particularly in urbanized states like Selangor, which continue to experience recurrent outbreaks despite ongoing government-led control efforts. This study explores how urban communities understand dengue, their attitudes toward prevention, and the actions they take to protect themselves. While the Health Belief Model (HBM) was used to structure the KAP (Knowledge, Attitude, Practice) questionnaire, the interpretation of findings is further supported by the Ideation Model. This approach provides a deeper understanding of how social norms, emotional cues, peer influence, and perceived community responsibility shape dengue-related behaviors. Applying ideation theory in this context, which is rarely done in Malaysian dengue studies, offers a more holistic view of the factors that drive or hinder preventive actions beyond individual cognition. The findings suggest that awareness alone is not enough; emotional and social motivators must also be addressed. This study emphasizes the need for culturally grounded education and collective community strategies to strengthen dengue prevention efforts. It offers practical guidance for health policymakers, practitioners, and community leaders in managing dengue hotspots in urban areas.

## 1. Introduction

*Aedes* mosquito-borne diseases have been a persistent threat to humans worldwide [1]. These diseases include malaria, dengue fever, and Zika virus and have caused much suffering and death over centuries [1,2]. Despite medicine and public health advancements [3], these diseases continue to pose substantial challenges, necessitating ongoing research, practical solutions, and global cooperation for effective control.

In response to this challenge, Malaysian authorities have implemented crucial measures to combat mosquito-borne diseases [4]. These initiatives include comprehensive efforts to control both the larvae and adult mosquitoes, health education campaigns, and enforcing actions [4–8]. In 2020, Malaysia began formally implementing the Integrated Vector Management (IVM) strategy as a national program for Aedes control, emphasizing cost-effective interventions and community engagement [9].

Previous studies on Aedes mosquito-borne diseases in urban communities have laid the foundation for understanding and addressing public health challenges. These investigations explored the factors influencing people’s knowledge, and attitudes, and practices (KAP) regarding malaria, dengue fever, and Zika virus and provided valuable insights into prevailing community perceptions. The results have informed public health interventions and educational campaigns aimed at enhancing community engagement and compliance with preventive measures [10–12].

However, the efficacy of these strategies relies heavily on community awareness and participation [5]. It is crucial that individuals understand these diseases and adopt practices and attitudes that can aid in preventing them. Education is a cornerstone in this fight [13], where it provides people with knowledge about these diseases and encourages good habits that can become a regular part of daily life. Furthermore, education is effective for dispelling myths and fostering a sense of urgency and cooperation among community members.

Despite the gravity of the issue, documented evidence regarding what the adult population in recurrent dengue hotspot areas knows about the disease, how they perceive it, and what preventive measures they take is lacking. Therefore, this study aimed to assess the KAP level related to dengue among adults in Hulu Langat, Selangor, Malaysia, which experiences recurrent dengue outbreaks. The main study goal was to assess the level of knowledge regarding dengue, its transmission, symptoms, and prevention measures in the Hulu Langat community. Additionally, we aimed to assess prevailing attitudes toward dengue prevention, including perceptions of the disease’s seriousness, personal responsibility, and the effectiveness of control measures. We hypothesized that better knowledge regarding dengue would correlate with more positive attitudes and more effective preventive practices.

This study adopted the Ideation Model as its primary conceptual framework to understand the multilevel influences on dengue prevention behaviors in recurring hotspot communities. The model emphasizes the interplay of cognitive, emotional, and social factors such as personal beliefs, interpersonal communication, and community norms in shaping behavior change. However, given the need for measurable constructs in the development of the KAP survey instrument, particularly for the attitude-related components, selected constructs from the Health Belief Model (HBM), including perceived susceptibility, severity, benefits, barriers, cues to action, and self-efficacy, were used to guide questionnaire development.

Although the HBM informed the instrument design and guided the analysis through its six core constructs, the interpretation of findings was grounded in the Ideation Model, particularly to explore broader sociocultural and interpersonal influences reflected in community responses. This integrated application provided both methodological structure through the HBM and richer theoretical insight through the Ideation Model, offering a more comprehensive understanding of the factors that influence dengue prevention behaviors. The approach has valuable implications for designing context-sensitive and community-based public health interventions.

## 2. Methodology

### 2.1. Ethics statement

The Ethical Review Committee for the National University of Malaysia Faculty of Health Science Department approved this study (ethics approval no. UKM PPI/111/8/JEP-2022–503). This approval was supported by the Ministry of Health Malaysia Medical Research & Ethics Committee (ethics approval no. NMRR ID-22–00314-FWY (IIR)). Implied consent was obtained via a written consent form attached to the questionnaire before initiating the survey. The respondents were informed verbally of the study objectives and aim to provide a clear explanation to them. We emphasized that the participation was voluntary and that the participants’ confidentiality and anonymity would be ensured.

### 2.2. Study design and setting

Selangor is the state with the highest reported dengue cases after Kuala Lumpur and Johor. The district of Hulu Langat is in southeastern Selangor, between Kuala Lumpur and Negeri Sembilan. Other than Petaling district, Hulu Langat recorded the most dengue fever cases in Selangor. Hulu Langat was selected based on the notable increase in dengue cases, which increased by 25.5% during the initial 7 weeks of 2022 compared to the corresponding period 2021 [14].

**Fig 1:**
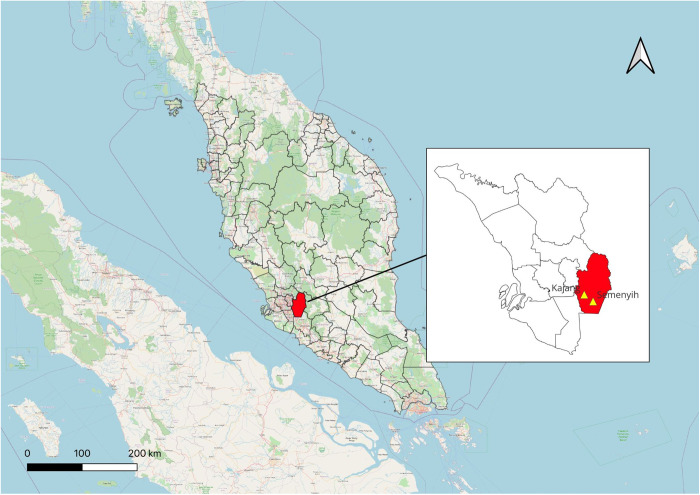
Map of the study area in Hulu Langat District, Selangor, Malaysia. Source: The map was created using QGIS. Administrative boundaries were sourced from GADM (CC BY 4.0), and the basemap features from OpenStreetMap (public domain). District shapefiles were downloaded from IGISMap. Coordinates were plotted in WGS 84. The final visual was enhanced using Canva. Map generated by the authors.

This cross-sectional study was conducted in March 2022 in Hulu Langat, Selangor, a state with recurrent dengue hotspot areas, specifically in Section 1 (Kajang) and Section 5 (Semenyih). In total, Section 1 has 850 households and Section 5 has 901 households, and these two locations are approximately 17.3 km apart. Both Kajang and Semenyih have geographic features, including proximity to shared amenities such as playgrounds, shops, shopping malls, and restaurants. This similarity contributes to their comparable appeal and accessibility. Overall, the cumulative number of dengue fever cases in Malaysia reported throughout 2022 increased by 150.7% (from 39,737–66,102 cases) compared to 26,365 in 2021 [15]. Malaysian Ministry of Health data demonstrated that Selangor consistently recorded the highest increase in dengue fever cases, with Hulu Langat being one of the most affected districts within the state [15]. The study locations are shown in Fig 1.

### 2.3. Sampling and sample size

Section 1 and Section 5 are located in the dengue cluster area and were proposed by the local authorities and district health authorities as the study sites. Respondents were recruited through purposive sampling to represent the general community of Sections 1 and 5. We used a sample size calculator (G*Power version 3.1), the details of F test as a test family, input parameters of 0.1, effect size f, 5% margin of error, and power of 0.8 to determine that the minimum recommended sample size was 125. Considering a 20% non-response rate, the final sample size was 146. Thus, the minimum number of respondents required for the survey from each section was 75. Based on the total number of residents in both sections, this study recruited the heads of households aged > 18 years, had been residing for >6 months, and were willing to commit to this study. The household heads were chosen as they represent the household behavior and influence its health practices [16]. Furthermore, household-level vector control is a fundamental strategy for preventing dengue fever [17].

### 2.4. Study instrument

The questionnaire used in this study was adapted from a previous study [13] that applied the Health Belief Model (HBM) to assess knowledge, attitudes, and preventive practices related to dengue. In the present study, however, the Ideation Model served as the overarching conceptual framework, providing a broader perspective to examine the interplay between individual cognition, interpersonal influences, and social norms. While the instrument design initially focused on four key HBM constructs—perceived susceptibility, severity, benefits, and barriers—for reasons of practicality and relevance, the analysis later incorporated all six constructs as cues to action and self-efficacy emerged during data interpretation. The overall adaptation process was also guided by preliminary needs assessments and community discussions conducted during the early phase of the study. For example, knowledge items were revised to reflect local misconceptions and behavioral patterns, while practice items were tailored to capture actions commonly adopted in recurrent dengue outbreak areas. This approach ensured conceptual alignment with the Ideation Model, while maintaining methodological rigor through the integration of established HBM constructs where applicable.

Four public health specialists reviewed the questionnaire content validity. The face validity of the questionnaire was revised based on the specialists’ feedback and further evaluated through a pilot test involving 30 respondents from other Hulu Langat localities who did not participate in the main study. These respondents provided feedback on the sentence structure, wording, comprehensibility, language suitability, and time required to complete the survey. The questionnaire was amended based on this feedback. The final structured questionnaire was printed in the Malay language to ensure comprehensibility for the respondents and consisted of Part A: sociodemographic data and history of dengue fever; Part B (1): knowledge of Aedes and dengue; Part B (2): knowledge of breeding sites and preventive practices; Part C: attitude constructs based on the HBM; and Part D: dengue preventive practices.

Attitude was measured using a 5-point Likert scale (1 = strongly disagree, 5 = strongly agree). Some attitude questions were reverse-coded to account for different response tendencies. For these reverse-coded questions, a lower score on the Likert scale (e.g., 1) indicated a more positive attitude, while a higher score (e.g., 5) indicated a more negative attitude. Consistency in the overall attitude score was ensured by adjusting the responses to these reverse-coded or negative questions during data analysis (i.e., a score of 1 was converted to 5, 2–4, 3–3, 4–2, and 5–1) before the scores were summed. This approach allowed us to maintain uniformity, where higher scores consistently reflected more positive attitudes regarding dengue prevention.

The overall reliability of the questionnaire was confirmed with a Cronbach alpha value of 0.834, indicating good internal consistency.

### 2.5. Data analysis

#### 2.5.1. Scoring for KAP.

- **Knowledge:** The respondents answered a set of knowledge-based questions on dengue and Aedes mosquitoes. Each correct response was awarded 1 point, and incorrect or “don’t know” answers were awarded 0 points. Each respondent’s total knowledge score was calculated as the sum of all correct answers. The maximum possible knowledge score was 40.
- **Attitude:** Attitudes regarding dengue prevention were assessed using 24 Likert scale questions ranging from 1 (strongly disagree) to 5 (strongly agree). Higher scores indicated more positive attitudes. For reverse-coded or negative questions, where a lower score represents a more favorable attitude, scores were adjusted during to maintain consistency. The total attitude score was calculated by summing the adjusted responses across all questions. With 24 questions, the possible score range was 24–120. The mean attitude score was computed to assess the overall attitude regarding dengue prevention.
- **Practice:** Preventive practices were measured through 11 yes/no questions. A “yes” response was awarded 1 point, and a “no” response was awarded 0 points. The total practice score was calculated by summing all “yes” responses, where higher scores indicated better engagement in dengue preventive behaviors.

#### 2.5.2. Interpretation of scores.

The KAP scores were interpreted using a cut-off point of 75% following the approach used by Okello et al. [18], which was adapted from Bloom’s classification framework [19]:

- Respondents with scores ≥75% were classified as having sufficient knowledge, positive attitudes, and good practices regarding dengue prevention.
- Respondents with scores < 75% were considered to have insufficient knowledge, negative attitudes, and poor practices regarding dengue prevention.

The knowledge, attitude, and practice components were scored separately. This classification enabled differentiation between respondents who were well-informed and engaged in preventive measures and those who were not.

#### 2.5.3. Statistical analysis.

The data were processed and analyzed using SPSS version 23. The respondents’ characteristics and responses were summarized using descriptive statistics [sample size (N) and percentages (%)]. Continuous variables were examined for normal distribution, and Kaiser-Meyer-Olkin (KMO) measures were applied to assess the suitability for factor analysis to Likert scale data. Quantitative data are presented as the mean ± standard deviation. Demographic groups were compared using Student’s *t*-tests. The associations between KAP variables were assessed using Spearman’s rank correlation coefficient, with statistical significance set at p < 0.05.

## 3. Results

### 3.1. Respondents’ demographic characteristics

**Fig 2:**
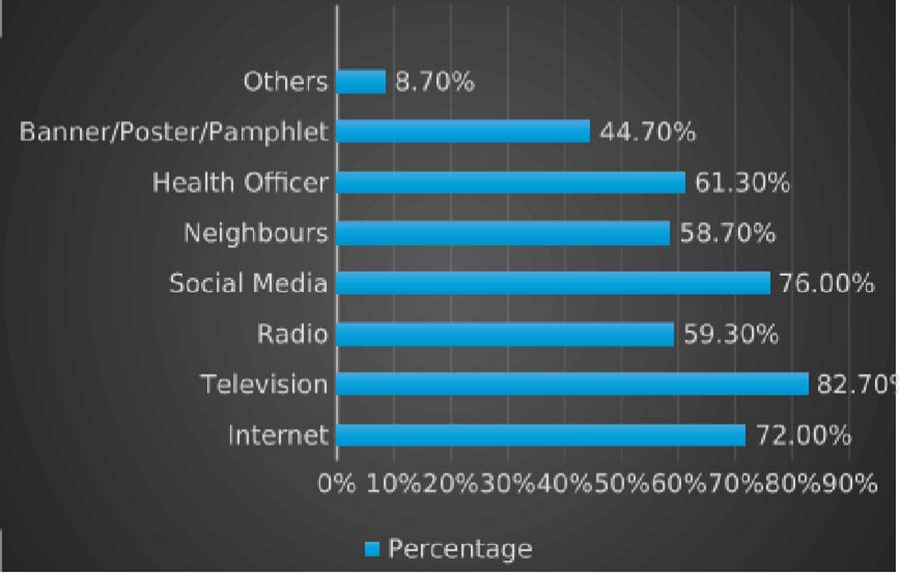
Respondents’ Information Sources on *Aedes* Mosquitoes and Dengue Fever. Percentages represent the proportion of respondents who cited each source of information.

The respondents’ mean age was 46.04 ± 12.067 years. Most respondents were male (53.5%) and Malay (90.0%). The majority (84.7%) were married, with a history of dengue fever recorded either by themselves (31.3%) or by family members (38.0%). In terms of education level, the respondents reported a wide range of academic backgrounds. More than half (56.2%) had completed the Sijil Pelajaran Malaysia (SPM), followed by 20.5% with a diploma, 16.4% with a bachelor’s degree, 5.5% with a master’s degree, and 1.4% with a PhD (results not shown). Regarding sources of dengue knowledge, television was the most common (82.7%), followed by social media (76.0%) and internet searches (72.0%). The least common source was banners, posters, or pamphlets (44.7%) (Fig 2). Fig 2 presents an overview of the various information sources on dengue fever reported by the respondents, highlighting general information dissemination patterns in the community.

### 3.2. Knowledge regarding *Aedes* mosquitoes, dengue, breeding spots, and prevention

The data indicated an overall insufficient knowledge among the respondents, where the respondents’ mean knowledge score was 22.54 ± 2.828. Specifically, 61.3% of respondents had insufficient knowledge. All respondents knew that the *Aedes* mosquito is black with white stripes. However, only 32.2% of the respondents knew that dengue fever is caused by a virus. Up to 97.3% of the respondents know that *Aedes* mosquitoes lay eggs in uncovered and clear water in containers. Up to 19.2% of the respondents provided the correct answer regarding the duration mosquito eggs can survive without water. Only 13% of the respondents provided the correct answers to the question on the active biting times of *Aedes* mosquitoes (Table 1).

**Table 1:** Frequency distribution of knowledge on *Aedes* and dengue (n = 146).

Knowledge questions	Correct answer	N (%)
Does a virus cause dengue fever?	Yes	47 (32.2)
How does dengue fever spread to other humans?	Mosquito bites	144 (98.6)
How long does it take for *Aedes* mosquito eggs to become adult *Aedes* mosquitoes?	7 days	55 (37.7)
Where do *Aedes* mosquitoes lay their eggs?	Uncovered clear water containers	142 (97.3)
What type of water can cause *Aedes* mosquitoes to lay eggs?	Clear	119 (81.5)
How long can mosquito eggs survive without water?	3–6 months	28 (19.2)
What color are *Aedes* mosquito eggs?	Black	40 (27.4)
What color are *Aedes* mosquito body parts?	Black with white stripes	134 (91.8)
When is the active biting time for mosquitoes?	6–8 a.m. and 7–9 p.m.	19 (13.0)
Choose the correct life cycle of *Aedes* mosquitoes	eggs – larvae – pupae – adults	69 (47.3)

Table 2 presents the data on the extent of knowledge regarding vector breeding sites and preventive practices. Insufficient knowledge was recorded regarding the steps to control and prevent mosquitoes from laying eggs, where only 30.1% of the respondents knew to treat water storage areas with mosquito larvicide. Up to 41.1% of the respondents knew to properly store all unused potential water containers. Most respondents had sufficient knowledge on the following steps that should be taken during a dengue fever outbreak: apply mosquito repellent (86.3%); keep doors and windows closed during mosquito active times (99.3%); wear bright colors, long sleeves, and long trousers (72.6%); use electric mosquito repellent or coils (92.5%); use mosquito nets (90.4%); and search for breeding sites and destroy them (97.9%). Regarding chemical/biological control measures, only 44.5% of the respondents allowed the authorities to spray inside and outside the residence and 40.4% knew to open all windows and doors during outdoor mosquito spraying.

**Table 2:** Frequency distribution of knowledge on breeding sites and the prevention (n = 146).

Knowledge questions	N (%)
Where can *Aedes* mosquitoes lay their eggs inside the house?	
a) Bathtub	125 (85.6)
b) Flowerpot saucers	144 (98.6)
c) Drain pan of refrigerator	127 (87.0)
d) Dish rack drainer	127 (87.0)
e) Water dispenser tray	120 (82.2)
Where can *Aedes* mosquitoes lay their eggs outside the house?	
a) Abandoned houses/buildings	135 (92.5)
b) Discarded tires	142 (97.3)
c) Piles of garbage	123 (84.2)
d) Water storage containers	145 (99.3)
e) Piles of items	124 (84.9)
f) Roof gutters	131 (89.7)
The following are steps to control and prevent mosquitoes from laying eggs. Please choose the correct statements:	
a) Properly dispose of trash in covered bins	144 (98.6)
b) Leave water in flowerpot saucers	75 (51.4)
c) Ensure that drainage channels flow freely and are not blocked	143 (97.9)
d) Treat water storage areas with mosquito larvicide	44 (30.1)
e) Store all unused containers that can hold water	60 (41.1)
f) Ensure that roof gutters are clear	144 (98.6)
What steps should be taken to prevent dengue virus infection during a dengue fever outbreak?	
a) Apply mosquito repellent to exposed body parts	126 (86.3)
b) Keep doors and windows closed when mosquitoes are active	145 (99.3)
c) Wear bright-colors, long sleeves, and long trousers when outdoors	106 (72.6)
d) Use electric mosquito repellents or mosquito coils when in dark, enclosed areas to repel adult *Aedes* mosquitoes inside the house	135 (92.5)
e) Use mosquito nets when sleeping	132 (90.4)
f) Searching for *Aedes* mosquito breeding sites and destroy them	143 (97.9)
What should be done when chemical/biological control measures are being used to eliminate *Aedes* mosquitoes?	
a) Allow health officer to inspect for mosquito larvae inside and outside the residence	138 (94.5)
b) Allow health officer to apply larvicide in potential mosquito breeding areas	142 (97.3)
c) Allow health officer to conduct mosquito spraying inside and outside the residence	65 (44.5)
d) Open all windows and doors during outdoor mosquito spraying	59 (40.4)
**Total knowledge score (mean ± SD)** **Sufficient knowledge** **Insufficient knowledge**	22.54 ± 2.828 58 (38.7) 88 (61.3)

### 3.3. Attitude toward dengue prevention based on the HBM

This study assessed respondents’ attitudes toward dengue prevention using constructs from the Health Belief Model (HBM). Specifically, attitudes were measured across six key domains: perceived severity, perceived susceptibility, perceived benefits, perceived barriers, self-efficacy, and cues to action—each representing beliefs that could influence personal responsibility and engagement in preventive behaviors.

**i. Perceived Severity**

A majority of respondents (87.0%) acknowledged that dengue fever could be fatal. However, 75.4% disagreed with the statement that a single *Aedes* mosquito bite could cause dengue, suggesting a low perceived risk from individual bites. Additionally, 47.9% disagreed that they could get infected if their neighbors had dengue, and 45.9% were unconcerned about prolonged fever, indicating limited awareness of indirect transmission risks.

**ii. Perceived Susceptibility**

Nearly half (47.3%) were unconcerned about leaving water containers outdoors, and 43.3% believed that maintaining a healthy lifestyle protected them from mosquito bites. Furthermore, 45.9% focused solely on indoor water containers, and 43.3% believed that repeated bites without falling sick indicated low personal risk.

**iii. Cues to Action**

While 93.2% said they would take action if their neighborhood was declared a hotspot, only 45.9% would rely on neighbors to clean the area after a case occurred nearby. In practice, fewer respondents were proactive: only 50.7% said they would help clean the surroundings, and just 40.4% reported checking for *Aedes* breeding sites weekly. Notably, 63.0% admitted not performing weekly checks.

**iv. Self-Efficacy**

About 59.6% said they would only act if instructed to do so. Additionally, 23.3% stated they would not take action even if neighbors were infected, and a similar proportion (23.3%) doubted the effectiveness of community-based prevention efforts. On a positive note, 95.2% indicated they would educate family members about *Aedes* mosquitoes.

**v. Perceived Barriers**

A significant number (62.3%) were unwilling to open doors or windows during fogging, and 50% believed fogging was harmful to health. Most respondents (84.2%) reported that family members did not assist in eliminating breeding sites, and 58.2% noted that homes with breeding sites were not fined, possibly reducing compliance motivation.

**vi. Perceived Benefits**

More than half (55.5%) agreed they would eliminate mosquito breeding sites if fines were imposed. About half of the respondents expressed interest in involving neighbors in search-and-destroy activities. Meanwhile, 41.1% believed fogging helped eliminate adult mosquitoes, and 43.2% were willing to educate their neighbors on dengue prevention.

These findings (Table 3) illustrate that while respondents were aware of the severity of dengue, perceived susceptibility and proactive preventive behaviors were still lacking. Understanding these belief patterns provides crucial insights into behavioral drivers and barriers, which can inform future dengue prevention strategies.

**Table 3:** Frequency distribution of respondents’ attitude scoring based on the HBM (n = 146).

Construct/statement	Disagree (%) (strongly disagree + disagree)	Neutral (%)	Agree (%) (agree + strongly agree)	KMO
**Perceived severity**				0.848
I don’t believe dengue fever can be deadly.	127(87.0)	4(2.7)	15(10.2)	
One *Aedes* mosquito bite can give me dengue fever.	110(75.4)	25(17.1)	11(7.8)	
I could get dengue fever if my neighbors had it.	70(47.9)	16(11.0)	60(41.1)	
Prolonged fever worries me.	67(45.9)	5(3.4)	74(50.7)	
**Perceived susceptibility**				0.853
I’m not concerned when I leave potential water containers outside.	69(47.3)	10(6.8)	67(45.9)	
Mosquito bites don’t affect me because I live a healthy lifestyle.	11(7.5)	7(4.8)	128(87.7)	
I only need to aware the water containers inside my house.	66(45.2)	8(5.5)	72(49.3)	
Even though I’ve been bitten by mosquitoes many times, I’ve never gotten sick, so I consider mosquito bites to have no effect on me.	10(6.9)	8(5.5)	128(87.7)	
**Perceived cues to action**				0.832
I tend to let my neighbors clean my house area when there’s a dengue case.	67(45.9)	13(8.9)	66(45.2)	
I will act when my neighborhood is declared a dengue fever hotspot.	7(4.8)	3(2.1)	136(93.2)	
I will participate in community efforts to clean the residential area to prevent dengue.	74(50.7)	8(5.5)	64(43.8)	
I will check for potential *Aedes* mosquito breeding sites once a week.	76(52.1)	11(7.5)	59(40.4)	
**Perceived self-efficacy**				0.836
I only take preventive measures and control mosquitoes if instructed to do so.	34(23.3)	25(17.1)	87(59.6)	
I don’t take any *Aedes* mosquito prevention measures, even if my neighbor gets sick from them.	4(2.7)	3(2.1)	139(95.2)	
I feel that community efforts to eliminate *Aedes* mosquitoes are no longer effective.	34(23.3)	25(17.1)	87(59.6)	
I will inform my family members about *Aedes* mosquitoes.	4(2.7)	3(2.1)	139(95.2)	
**Perceived barrier**				0.835
I won’t open doors and windows during mosquito fogging.	25(17.1)	30(20.5)	91(62.3)	
The smoke from mosquito fogging conducted by the authorities is harmful to health.	73(50.0)	32(21.9)	41(28.1)	
My family doesn’t help and support in finding and destroying *Aedes* mosquito breeding sites.	17(11.6)	6(4.1)	123(84.2)	
There are no fines if my house has *Aedes* mosquito breeding sites.	85(58.2)	15(10.3)	46(31.5)	
**Perceived benefit**				0.878
If fines are imposed, I will ensure my house is clean without *Aedes* mosquito breeding sites.	81(55.5)	11(7.5)	54(37)	
I will be enthusiastic if many neighbors join in the search and destruction of *Aedes* mosquito breeding sites.	52(35.6)	21(14.4)	73(50)	
Fogging helps kill adult *Aedes* mosquitoes.	60(41.1)	7(4.8)	79(54.1)	
I will assist in providing health education to neighbors.	63(43.2)	9(6.1)	74(50.7)	
**Total attitude (mean ± SD)**	69.70 ± 6.027			
**Negative attitude**	105 (71.9)			
**Positive attitude**	41 (28.1)			

### 3.4. Respondents’ prevention practices

The respondents exhibited a high prevalence of poor practices regarding protective measures against dengue, with 57.5% falling under the category of poor practice. Despite this, most respondents (89%) reported securing plastic trash bags and placing them in a bin as a good practice. However, adherence was significantly lower for other preventive actions. For example, only 43.8% of the respondents regularly empty and clean containers that collect water, and 47.9% made efforts to avoid storing containers that can trap water. Furthermore, there were low rates of practices such as ensuring proper drainage around the home (27.4%), using aerosol sprays indoors as needed (37.7%), and allowing fogging indoors (36.3%). Additionally, only 40.4% of the respondents checked for mosquito breeding spots weekly. The overall mean practice score was 6.16 ± 2.014, confirming that most respondents had poor practices regarding dengue prevention (Table 4).

**Table 4:** Frequency distribution of respondents’ prevention practices (n = 146).

Practice questions	N (%)
Secure plastic trash bags and place them in a bin.	130 (89.0)
Regularly empty and clean containers that collect water.	64 (43.8)
Avoid keeping containers that can trap water.	70 (47.9)
Ensure that valuable items stored behind the house do not collect water.	56 (38.4)
Participate in community clean-up events to help prevent dengue outbreaks.	70 (47.9)
Allow fogging indoors.	53 (36.3)
Ensure proper drainage around your home.	40 (27.4)
Check for mosquito breeding spots weekly.	59 (40.4)
Always keep water containers covered.	69 (47.3)
Use aerosol sprays indoors as needed.	55 (37.7)
Collect and dispose of water-collecting containers in the trash.	75 (51.4)
**Total practice score (mean ± SD)** **Poor practice** **Good practice**	6.16 ± 2.014 84 (57.5) 62 (42.5)

### 3.5. Analysis of KAP scores with respect to demographic characteristics

The analysis of demographic factors revealed interesting patterns in the community’s knowledge, attitudes, and practices regarding dengue prevention. Although females showed slightly higher mean knowledge and attitude scores than males, these differences were not statistically significant, indicating similar awareness levels across genders. Ethnic differences were apparent in practice scores, with Malay respondents demonstrating significantly higher practice scores than other groups, suggesting more active engagement in preventive behaviors within this population. Marital status also influenced attitudes; notably, single and divorced respondents had higher attitude scores compared to married and widowed individuals, which may reflect differing levels of health concern or social support. Education level appeared to be linked to knowledge, as respondents with higher education achieved better knowledge scores; however, those with lower education levels surprisingly reported higher attitude and practice scores. This pattern suggests that formal education may not be the sole driver of preventive behaviors and attitudes, highlighting the potential influence of community factors or targeted health messaging. Overall, these findings underscore the importance of tailoring dengue prevention efforts to specific demographic groups to enhance effectiveness. These results are summarized in Table 5.

**Table 5:** Comparison of KAP scores according to demographic variables (n = 146).

Demographic data	No.	Knowledge score		Attitude score		Practice score	
		(mean ± SD)	p-value	(mean ± SD)	p-value	(mean ± SD)	p-value
**Sex**			0.583		0.270		0.006*
Male	80	32.09 ± 4.28		69.63 ± 5.61		8.63 ± 1.75	
Female	70	33.06 ± 5.37		69.79 ± 6.51		7.63 ± 2.17	
**Race**			0. 148		0.367		0.018*
Malay	135	32.79 ± 4.34		69.46 ± 5.90		8.30 ± 1.98	
Chinese	5	32.00 ± 4.24		73.80 ± 6.49		5.60 ± 1.95	
Indian	9	29.00 ± 9.72		71.22 ± 7.58		7.56 ± 1.74	
**Age**			0.578		0.248		0.934
18–20 years	3	28.67 ± 4.51		73.00 ± 11.53		8.67 ± 3.22	
21–30 years	13	32.46 ± 4.12		69.69 ± 6.09		7.85 ± 1.95	
31–40 years	25	32.72 ± 2.67		67.32 ± 7.19		8.36 ± 1.80	
41–50 years	43	33.16 ± 6.83		70.16 ± 5.08		8.07 ± 2.04	
>50 years	66	32.36 ± 3.98		70.15 ± 5.80		8.18 ± 2.08	
**Marital status**			0.562		0.032*		0.057
Single	12	32.33 ± 3.37		72.50 ± 6.61		7.92 ± 2.07	
Married	127	32.70 ± 4.93		69.39 ± 5.92		8.27 ± 1.91	
Widowed	6	29.83 ± 5.38		66.33 ± 4.13		8.33 ± 1.97	
Divorced	5	32.20 ± 4.82		75.00 ± 5.34		5.80 ± 3.42	
**Education level**			0.700		0.300		0.004*
Primary	6	33.83 ± 13.45		73.17 ± 5.42		9.33 ± 1.51	
Secondary	53	31.79 ± 3.72		69.83 ± 6.07		8.74 ± 1.90	
Diploma	29	33.03 ± 4.76		68.52 ± 4.87		8.38 ± 1.89	
Degree	52	32.85 ± 4.68		70.25 ± 5.95		7.37 ± 2.02	
Other	10	32.70 ± 2.31		67.50 ± 8.82		7.90 ± 1.97	
**Residential property**			0.470		0.850		0.408
Owner	125	32.68 ± 4.77		69.96 ± 6.13		8.10 ± 1.97	
Renter	24	31.92 ± 5.23		68.17 ± 5.40		8.42 ± 2.30	

### 3.6. Correlation between respondents’ KAP scores

Spearman’s rho indicated the presence of a weak negative correlation between knowledge and practice (*r*_s_ = -0.163, p* *< 0.05). Attitude and practice were moderately negatively correlated (*r*_s_ = -0.310), and the p-value indicated a strong statistical significance (p < 0.01) (Table 6).

**Table 6:** Correlation between respondents’ KPA scores (n = 146).

Variable	Knowledge		Attitude	
	r-test	p-value	r-test	p-value
**Attitude**	-0.020	0.811	–	–
**Practice**	-0.163	0.046*	-0.310	0.001**

## 4. Discussion

This study initially adopted the Health Belief Model (HBM) to guide the development of the attitude component within the Knowledge, Attitudes, and Practices (KAP) questionnaire. HBM was selected due to its widespread use in health behavior research and its compatibility with structured survey instruments. Constructs such as perceived susceptibility, severity, benefits, and barriers provided a practical framework for assessing individual beliefs related to dengue prevention. However, HBM was applied solely for instrumental purposes during questionnaire design and did not serve as the main interpretive framework for analyzing community behavior.

As the data analysis progressed, it became evident that the HBM alone could not adequately explain the complex behavioral patterns observed among residents in this high-risk urban setting. Despite high levels of knowledge regarding dengue transmission, preventive practices remained inconsistent. Many participants expressed low personal responsibility, expecting local authorities or neighbors to take the lead in prevention efforts. These findings highlighted the limitations of individual-level models such as the HBM in capturing the broader social and emotional dynamics that influence behavior.

To address these limitations, the Ideation Model was adopted as the primary analytical lens. Unlike the HBM, which focuses narrowly on individual cognition, the Ideation Model offers a more comprehensive framework by incorporating emotional, social, and contextual determinants of behavior. It emphasizes the influence of interpersonal communication, perceived social norms, peer support, and collective efficacy—elements that emerged prominently from the study’s findings. For instance, the perceived lack of shared responsibility and low confidence in community action are more effectively interpreted through the Ideation Model’s constructs, which account for the role of social interaction and emotional drivers in shaping preventive behaviors.

The adoption of the Ideation Model for interpreting the findings led to several critical insights. It became clear that dengue prevention in recurrent hotspot areas cannot rely solely on knowledge dissemination or individual motivation. Instead, communication strategies must also foster emotional engagement, strengthen peer influence, and build a sense of collective identity around preventive practices. The study found that family members, neighbors, and local leaders served as important cues to action—emphasizing the need for interventions that promote community-wide responsibility and social cohesion in dengue control efforts.

Public health education remains essential in raising awareness and shaping attitudes toward the severity and risks of recurrent dengue outbreaks. However, knowledge alone does not automatically translate into positive attitudes or sustained preventive practices. Several studies have reported that while individuals may have adequate knowledge about dengue, they often fail to adopt consistent prevention behaviors [20,21]. This underscores the importance of moving beyond traditional educational campaigns to interventions that address behavioral motivation and community-level engagement.

This study assessed KAP related to dengue among 146 respondents in selected localities in Hulu Langat. The results revealed significant gaps, consistent with prior research conducted in other Malaysian settings [5]. The mean knowledge score was 27.47 ± 4.83, indicating a moderate level of understanding with considerable variability. Only 38.7% of respondents demonstrated sufficient overall knowledge (Table 2), 27.3% showed positive attitudes (Table 3), and 44% reported good preventive practices (Table 4). These findings suggest persistent challenges in engaging the community effectively, particularly in areas facing repeated dengue outbreaks. Moreover, the findings differed from studies in other parts of Selangor [13,20], suggesting that specific contextual or environmental barriers may influence community response in this recurrent hotspot.

Further analysis revealed that while respondents had good knowledge of certain *Aedes* mosquito characteristics, such as body color, breeding sites, and transmission methods, gaps remained in more fundamental areas. Few participants were aware that dengue is caused by a virus, the mosquito’s full life cycle, or the color of its eggs. These results align with previous studies showing that although the public often feels well-informed through sources like television, social media, and the internet, comprehension remains partial and inconsistent [17,22–24].

Fig 2 illustrates that most respondents received dengue-related information through mass and digital media. However, the effectiveness of these platforms in fostering accurate understanding and behavior change remains questionable. Passive information exposure does not necessarily translate into knowledge retention or behavioral adoption. Prior research supports this, showing that media campaigns may increase awareness but have limited influence on actual practices unless combined with efforts to boost self-efficacy and active engagement [25,26].

Interestingly, traditional materials such as banners, posters, and pamphlets were among the least cited sources of information. This raises concerns about whether such methods are being underutilized or perceived as ineffective. However, some studies affirm their continued relevance when integrated with other forms of communication. For example, Barik et al. [27] found that print materials can be impactful when paired with video content or interactive activities. Similarly, Ali et al. [28] and findings from Venezuela [29] demonstrated that community-based, face-to-face engagement tends to yield more significant behavior change than passive media alone. These findings collectively suggest that a hybrid approach combining mass media, traditional outreach, and community participation is needed to improve both knowledge uptake and preventive practices in recurrent hotspot areas.

There are numerous potential breeding sites for *Aedes* mosquitoes in both indoor and outdoor environments, especially in areas with clean, stagnant water. Every individual must understand that *Aedes* mosquitoes can breed even in minimal amounts of water [30]. In line with findings from studies in Nepal [31] and Malaysia [32], our study revealed that respondents generally possessed a good level of knowledge regarding mosquito breeding sites, indicating a solid awareness of the environmental factors contributing to dengue transmission. However, a large proportion of respondents strongly agreed that prevention efforts should focus mainly on water containers outside the home, reflecting an externalized sense of susceptibility. Despite this knowledge, preventive practices remained insufficient. Only about half of the participants actively avoided storing water-trapping containers, and just 26.7 percent took additional steps such as ensuring proper drainage around their homes. This highlights a consistent pattern: knowledge alone does not necessarily translate into proactive behavior, a theme also documented in earlier studies [33,34].

These findings underscore the relevance of using the Ideation Model to better understand and interpret the cognitive, emotional, and social factors that influence dengue-related behaviors. While the Health Belief Model (HBM) provided the theoretical basis for certain attitude constructs, particularly perceived susceptibility, severity, benefits, and barriers, the broader framework of ideation was more appropriate for capturing the full complexity of behavior change. The Ideation Model incorporates not only individual cognition and beliefs but also social norms, self-efficacy, interpersonal communication, and emotional drivers, all of which are especially relevant in community-based settings.

One such example is the issue of fogging operations. The Malaysian health authorities have acknowledged the logistical challenges of conducting fogging inside individual premises [35], although vector control through fogging remains an essential strategy to reduce *Aedes* populations [36]. In our study, many respondents demonstrated adequate knowledge about the purpose of fogging and were aware that windows and doors should remain open during such operations. Nevertheless, a conflicting pattern emerged. Most respondents also expressed reluctance to keep their homes open during fogging due to health concerns, reflecting a classic case of perceived barriers overriding knowledge.

This contradiction highlights deeper emotional and trust-related dimensions that cannot be fully explained by cognitive models alone. Previous studies [5,37,38] have similarly reported skepticism about the effectiveness of fogging and concerns about its safety. For instance, one Malaysian study [39] linked frequent and inconsistent fogging to a rise in dengue cases, which in turn undermined community trust in the intervention. Conversely, other research [5] showed that communities actively requested fogging during outbreaks. This variability in acceptance demonstrates the need for health authorities to go beyond simply disseminating information. Instead, clear and empathetic communication strategies that address both informational gaps and emotional concerns are essential to improve public confidence in fogging and other vector control measures [40].

Social influences, another core element of the Ideation Model, also played a significant role in shaping behaviors. Family members and neighbors were identified as important sources of influence regarding dengue prevention practices [41]. Within the home, family often reinforces the adoption of healthy behaviors such as covering water containers and eliminating breeding grounds [42], while neighbors can either facilitate or hinder these practices through shared community norms [43]. In this study, many respondents perceived their families as less supportive in identifying and eliminating breeding sites. In contrast, the community viewed their neighbors more positively, seeing them as potential collaborators. However, respondents frequently reported low self-efficacy, particularly in terms of believing that collective community action could effectively reduce mosquito populations. This finding contrasts with earlier research, which suggested a higher sense of community ownership and responsibility for dengue control [43]. These gaps in perceived support and self-efficacy highlight the necessity of community empowerment strategies that build not only knowledge but also confidence and collective motivation, which are key elements of the ideation framework.

Moreover, Becker’s interpretation of the Health Belief Model (HBM) [44] emphasizes that behavior is driven by perceived vulnerability and the perceived seriousness of the disease. Applying this perspective to our study population, especially in a locality frequently affected by outbreaks, makes it clear that increasing awareness alone is insufficient without also fostering emotional readiness and self-belief. Respondents with limited understanding of dengue risk were less likely to engage in preventive actions. The most commonly reported practice was using proper trash bags and bins, a relatively passive behavior. Meanwhile, more proactive behaviors, such as checking and draining containers weekly, were not consistently practiced, even among those with high levels of knowledge [45,46]. This discrepancy echoes findings from other Malaysian studies [15,22] and reflects the critical gap between knowing what to do and feeling motivated, supported, and able to do it consistently.

A key insight from this study is the persistent disconnection between knowledge and actual behavior. While participants were well-informed about dengue prevention, including eliminating standing water and covering containers, the practical implementation of these behaviors was sporadic. This mismatch, also noted in global literature, has been consistently reported in the Malaysian context [15,22]. The findings reinforce the argument that health interventions must move beyond education to address the behavioral, emotional, and social dimensions of change. The Ideation Model proves especially relevant in this regard, offering a holistic lens through which to understand not just what people know, but also what they think, feel, and believe, and how these factors translate into action.

In conclusion, this study reinforces the complex interplay between knowledge, attitudes, and behaviors in dengue prevention. By integrating the Ideation Model with selected constructs from the Health Belief Model, we developed a robust interpretive framework that accounted for the multiple drivers of behavior in a recurrent dengue hotspot setting. Our findings suggest that successful intervention requires more than just providing information. It must also nurture self-efficacy, address emotional and social barriers, and foster a sense of shared responsibility within the community. Only by tackling these multi-layered factors can we hope to create sustainable behavior change and reduce dengue transmission in high-risk areas.

## 5. Conclusion

Although the Health Belief Model (HBM) guided the development of attitude-related items in our KAP questionnaire, the broader interpretation of our findings benefits significantly from the Ideation Model. The HBM effectively captures individual cognitive constructs such as perceived susceptibility, severity, and self-efficacy. However, our findings suggest that understanding dengue prevention behaviors, particularly in urban communities affected by recurrent outbreaks, requires moving beyond individual perceptions alone.

The Ideation Model, although not directly embedded in the questionnaire design, offers a valuable interpretive framework for exploring the social, emotional, and contextual influences on preventive behavior. Factors such as family dynamics, peer influence, perceived social norms, and collective responsibility play critical roles in shaping how individuals engage in dengue prevention. While conventional KAP studies primarily focus on individual knowledge and risk perception, this study extends the interpretation to include emotional, normative, and interpersonal influences — dimensions often underexplored in dengue research, particularly within the Malaysian context.

By incorporating this ideation-informed perspective, our study contributes a more nuanced lens to the public health discourse. It emphasizes the importance of integrating personal beliefs with broader sociocultural dynamics. This approach is especially relevant in endemic areas where traditional models such as the HBM may not fully explain or sustain behavioral change. Our findings indicate the need for future dengue interventions that are not only shaped by individual-level constructs but also grounded in community structures, collective efficacy, and culturally responsive communication strategies. Integrating the Ideation Model into dengue prevention efforts presents a promising direction for achieving more effective and sustainable public health outcomes.
